# Supplementary material for: Rhinacanthin-C but Not -D Extracted from Rhinacanthus nasutus (L.) Kurz Offers Neuroprotection via ERK, CHOP, and LC3B Pathways
Source: Pharmaceuticals (Basel). 2022 May 20;15(5):627. doi: 10.3390/ph15050627 (PMC9145051; doi:10.3390/ph15050627)
Supplement: Supplementary file 1 [file pharmaceuticals-15-00627-s001.zip › pharmaceuticals-1677432-supplementary.pdf]

Supplementary data

A

Rhinacanthin C

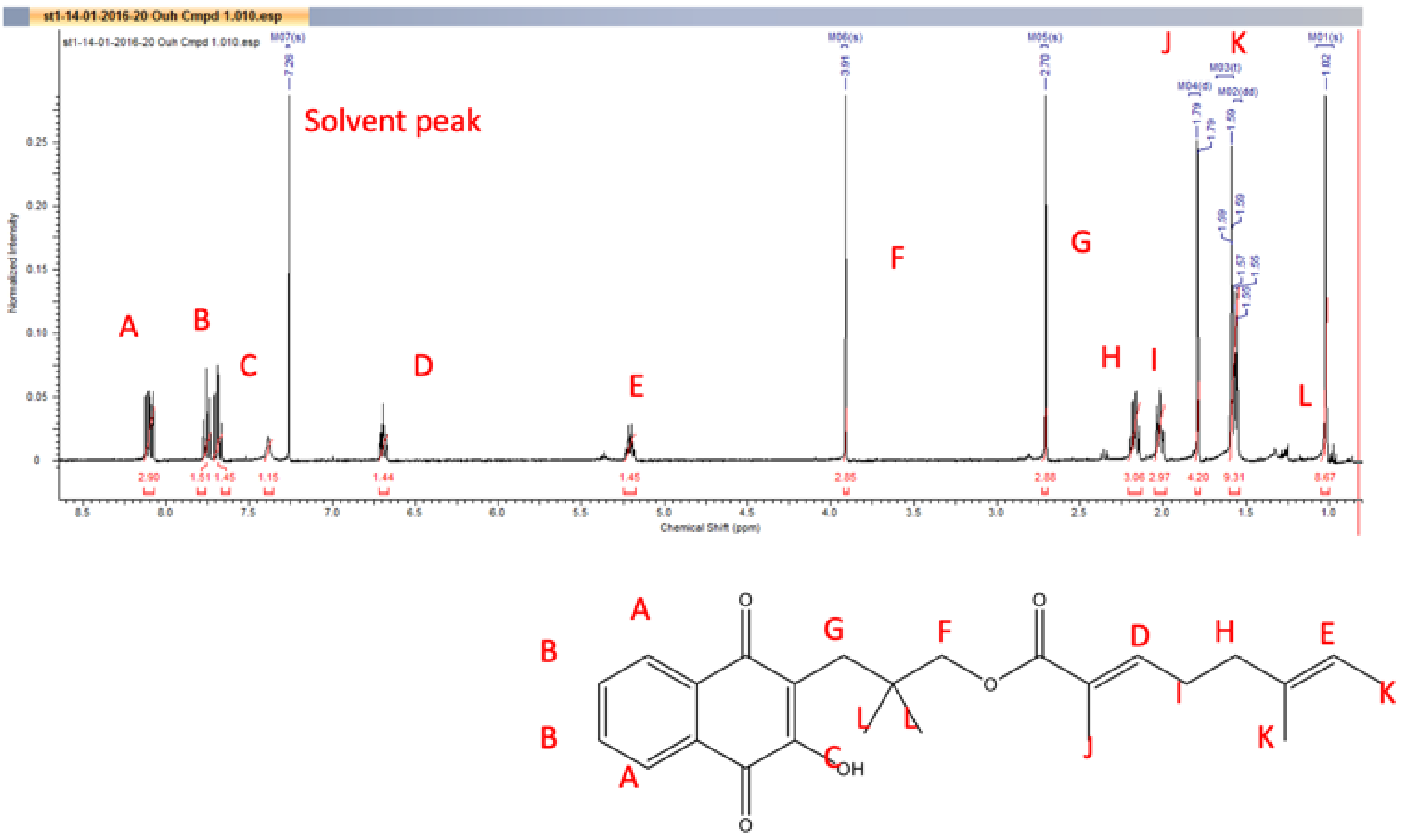

Supplementary Figure S1. **A** ^{1}H NMR spectrum (1-dimensional) of Rhinacanthin C plotted as signal intensity vs. chemical shift. Full plot.

B

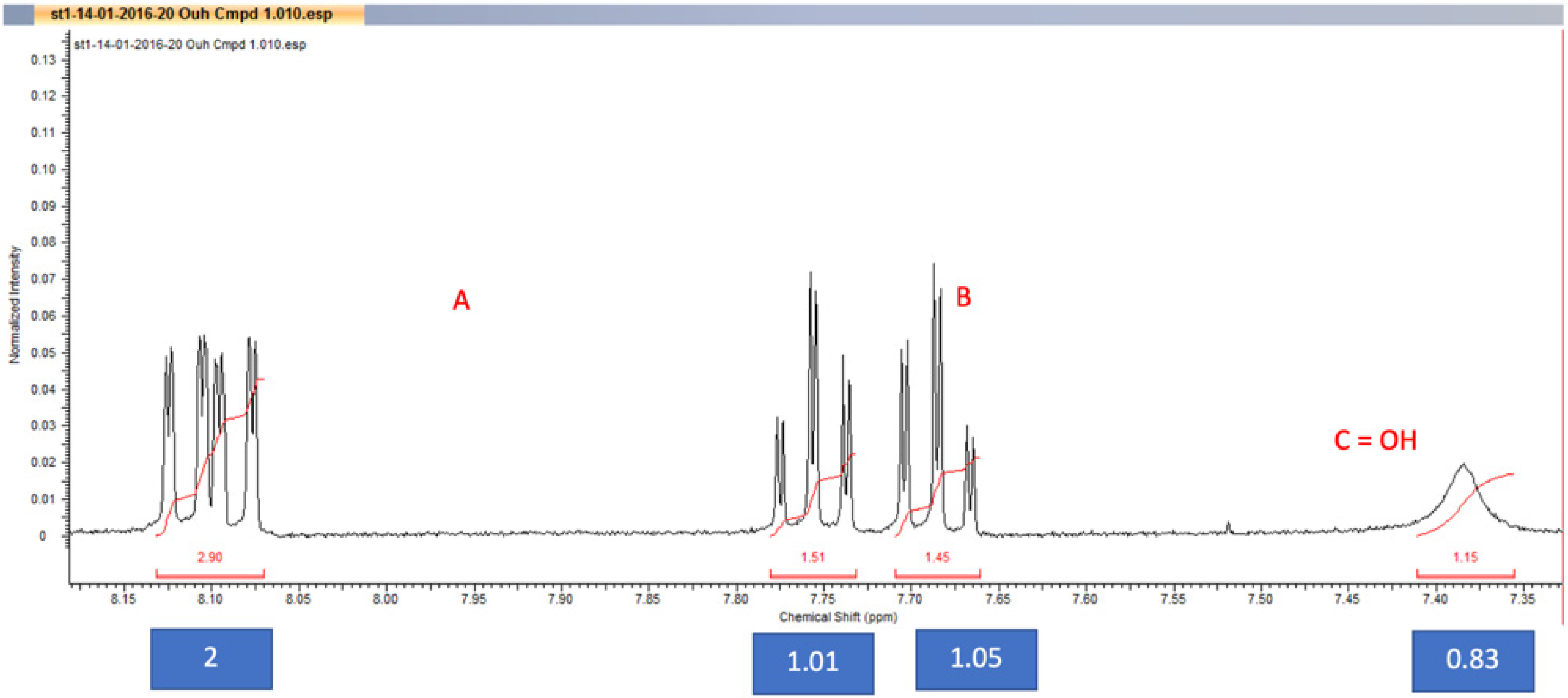

Supplementary Figure S1. **B** ^{1}H NMR spectrum of Rhinacanthin C, highlighting the aromatic portion of Rhinacanthin C (A-B from Sup. Fig. 1A) plus the -OH group. (C from Sup. Fig.1A).

C

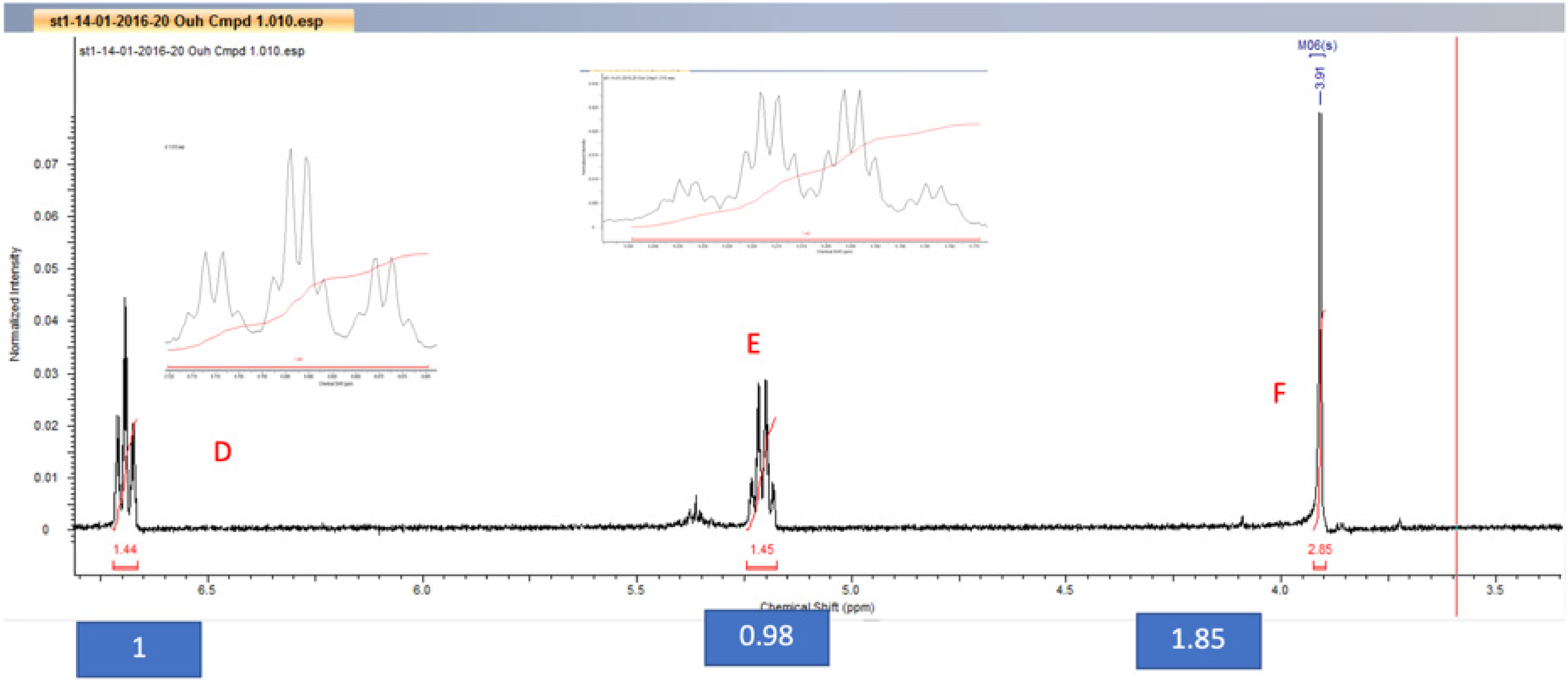

Supplementary Figure S1 **C.** ^{1}H NMR spectrum of Rhinacanthin C, highlighting the carbon atoms D, E & F from Sup. Fig. 1A

D

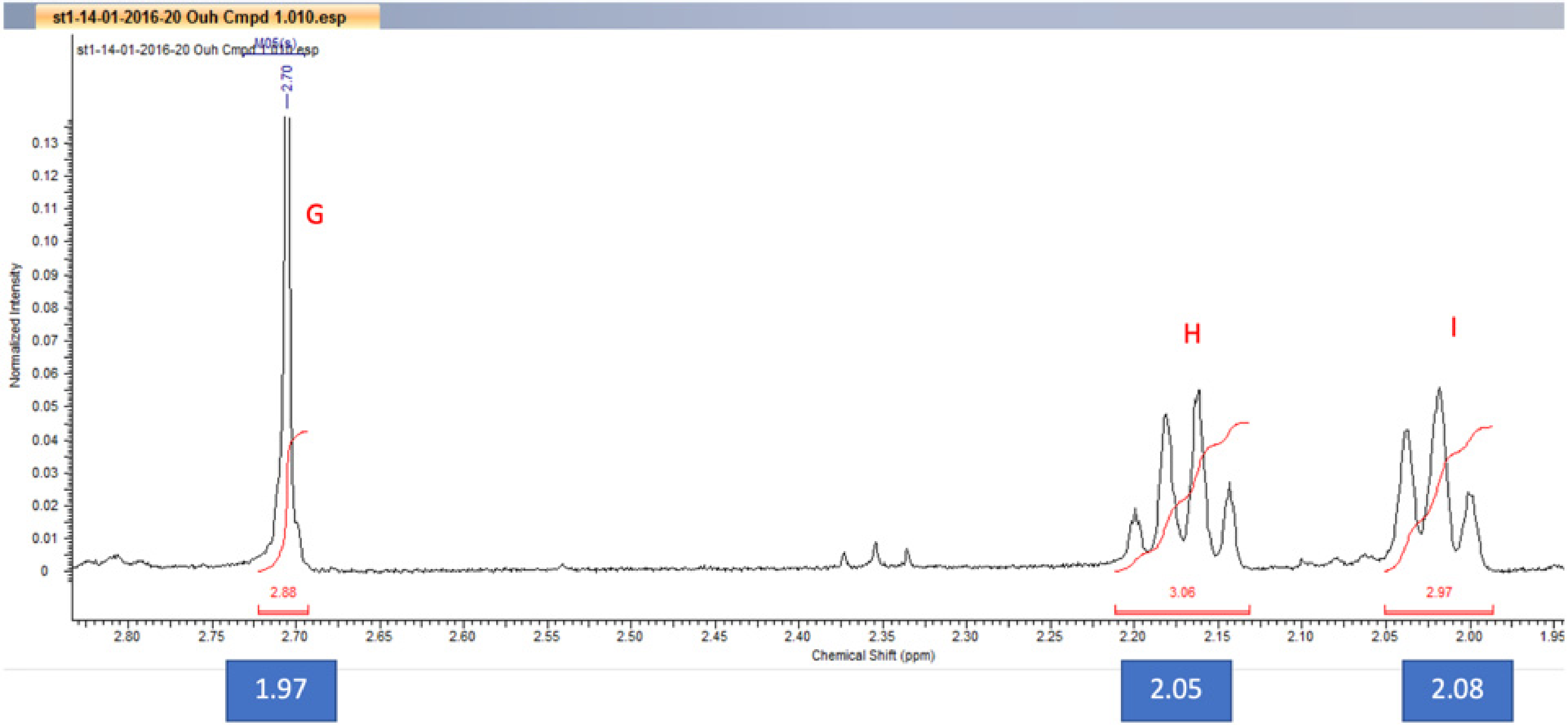

Supplamentary Figure S1 **D.** ^{1}H NMR spectrum of Rhinacanthin C, highlighting the carbon atoms G, H & I from Sup. Fig. 1A

A

Rhinacanthin-D

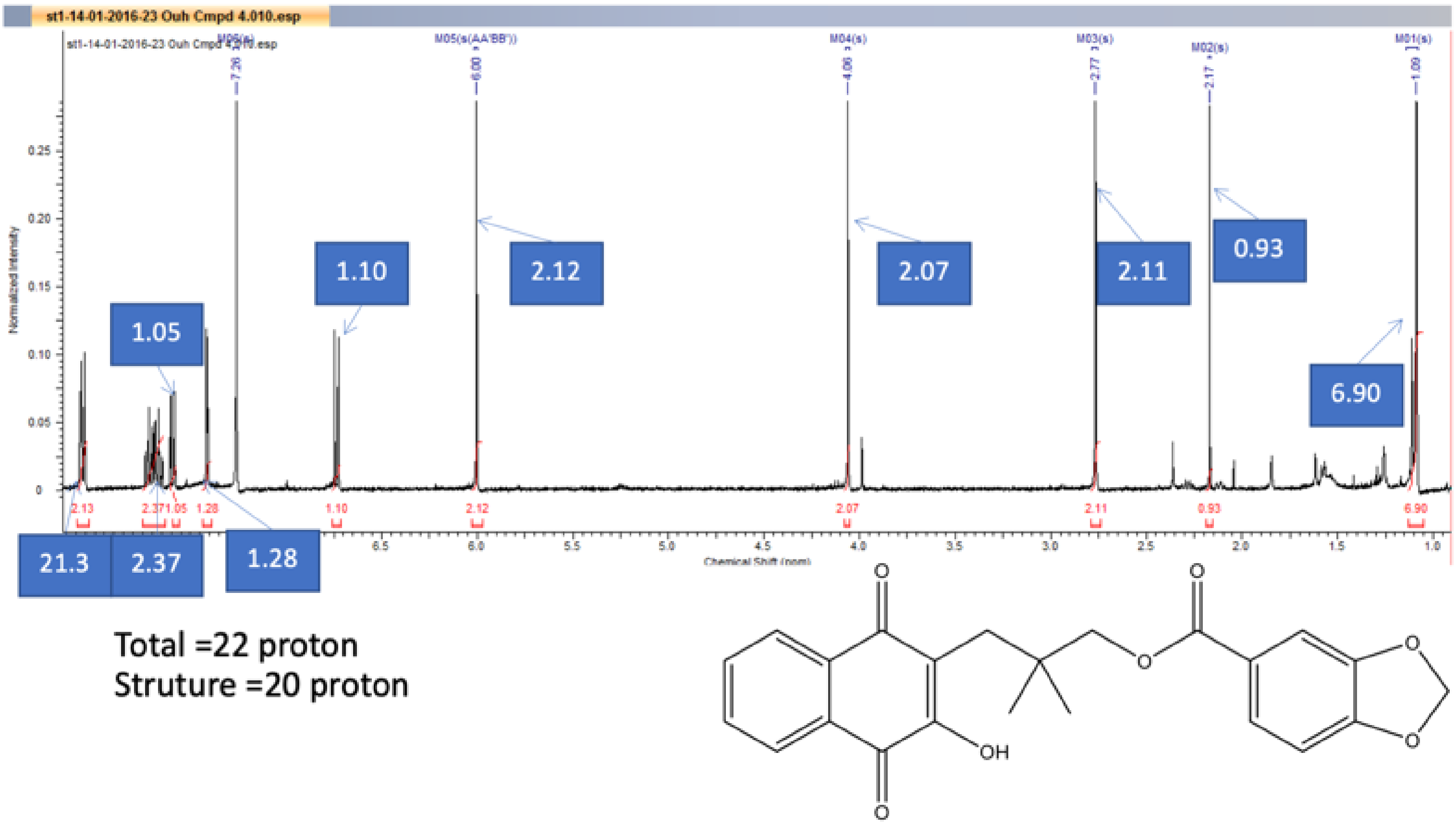

Supplementary Figure S2**A**. ^{1}H NMR spectrum of Rhinacanthin D, plotted as signal intensity vs. chemical shift. Full plot.

B

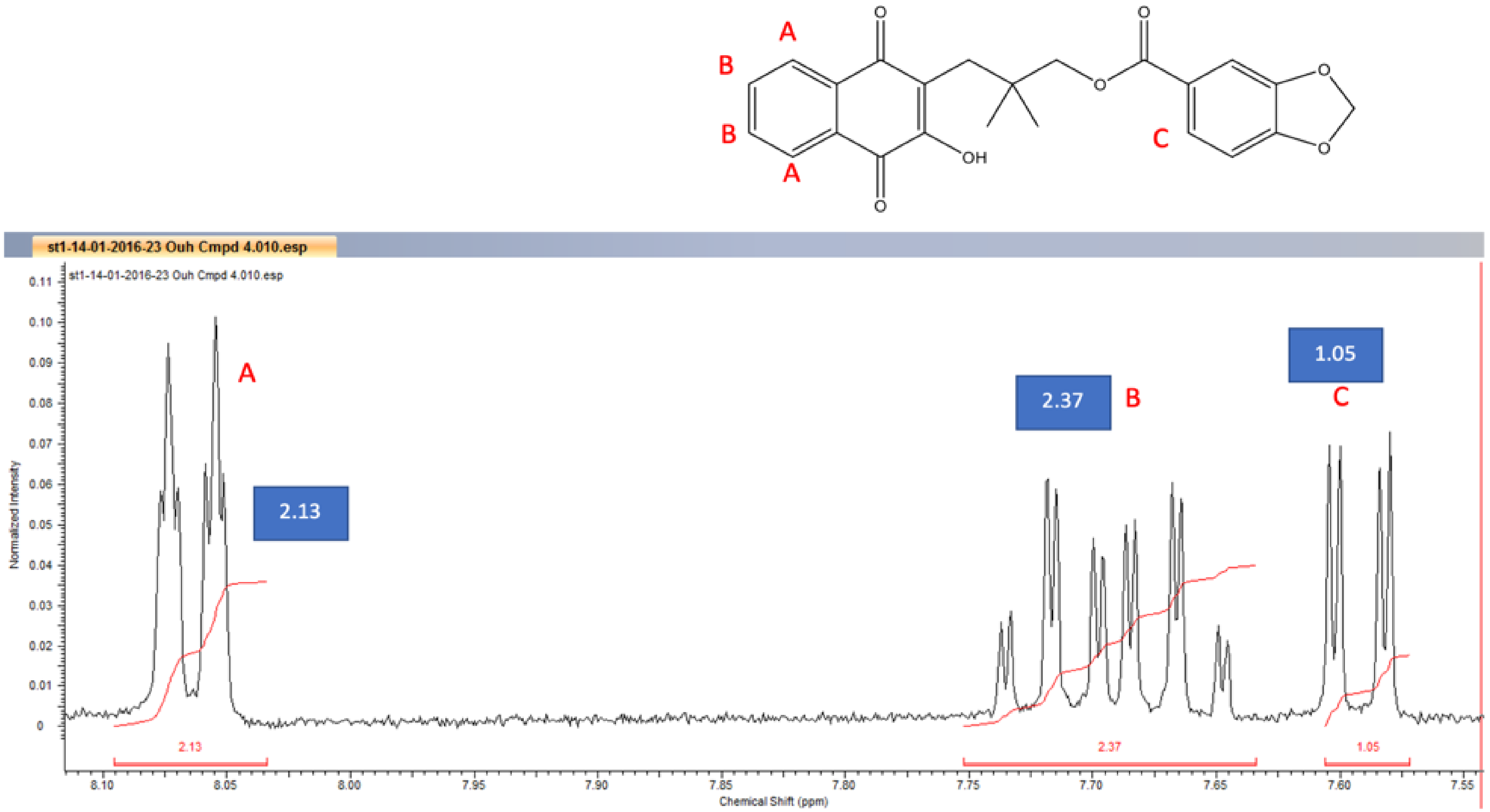

Supplementary Figure S2**B.** ^{1}H NMR spectrum of Rhinacanthin C, highlighting the aromatic portion of Rhinacanthin D

C

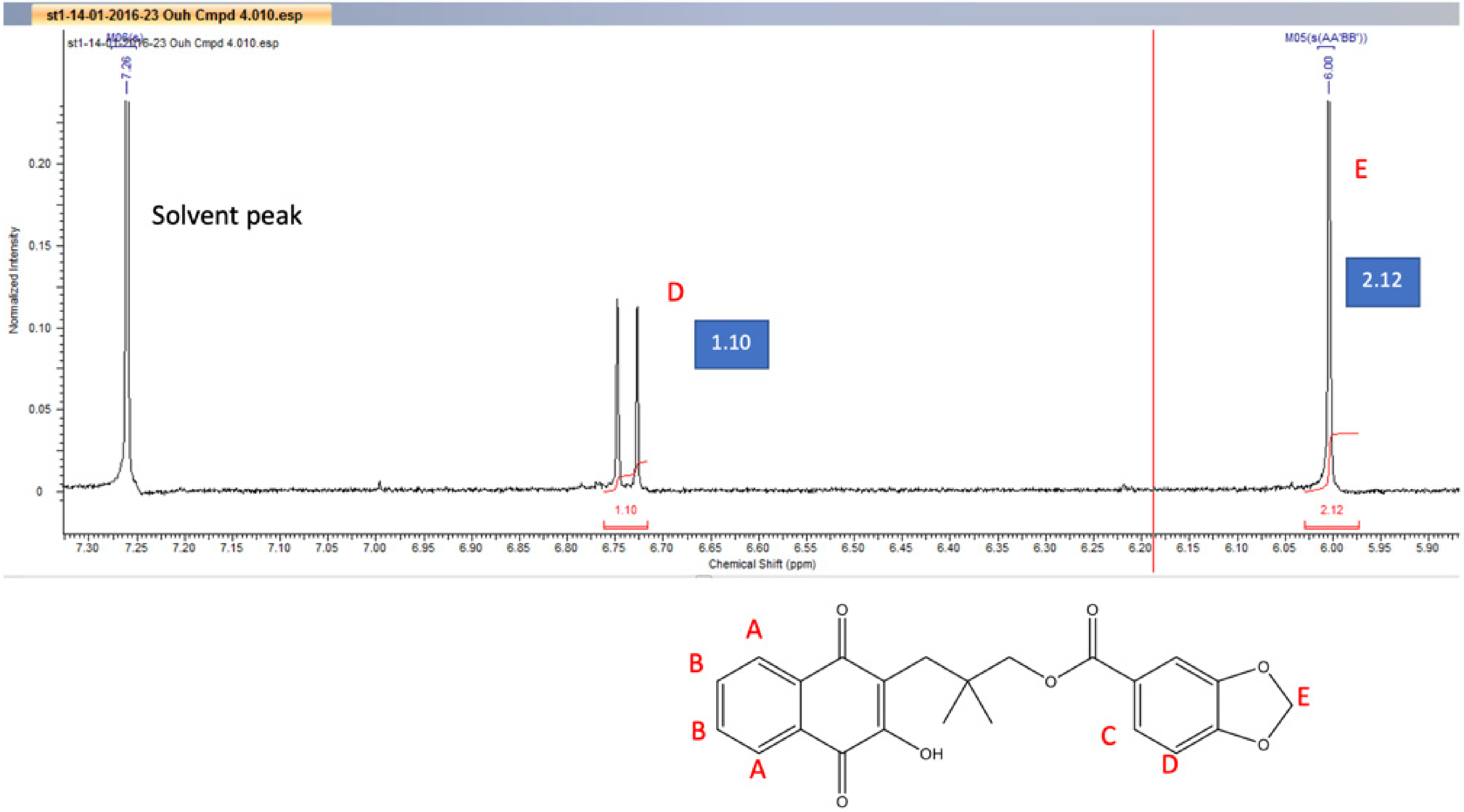

Supplementary Figure S2**C.** ^{1}H NMR spectrum of Rhinacanthin C, highlighting the aromatic portion of Rhinacanthin D

D

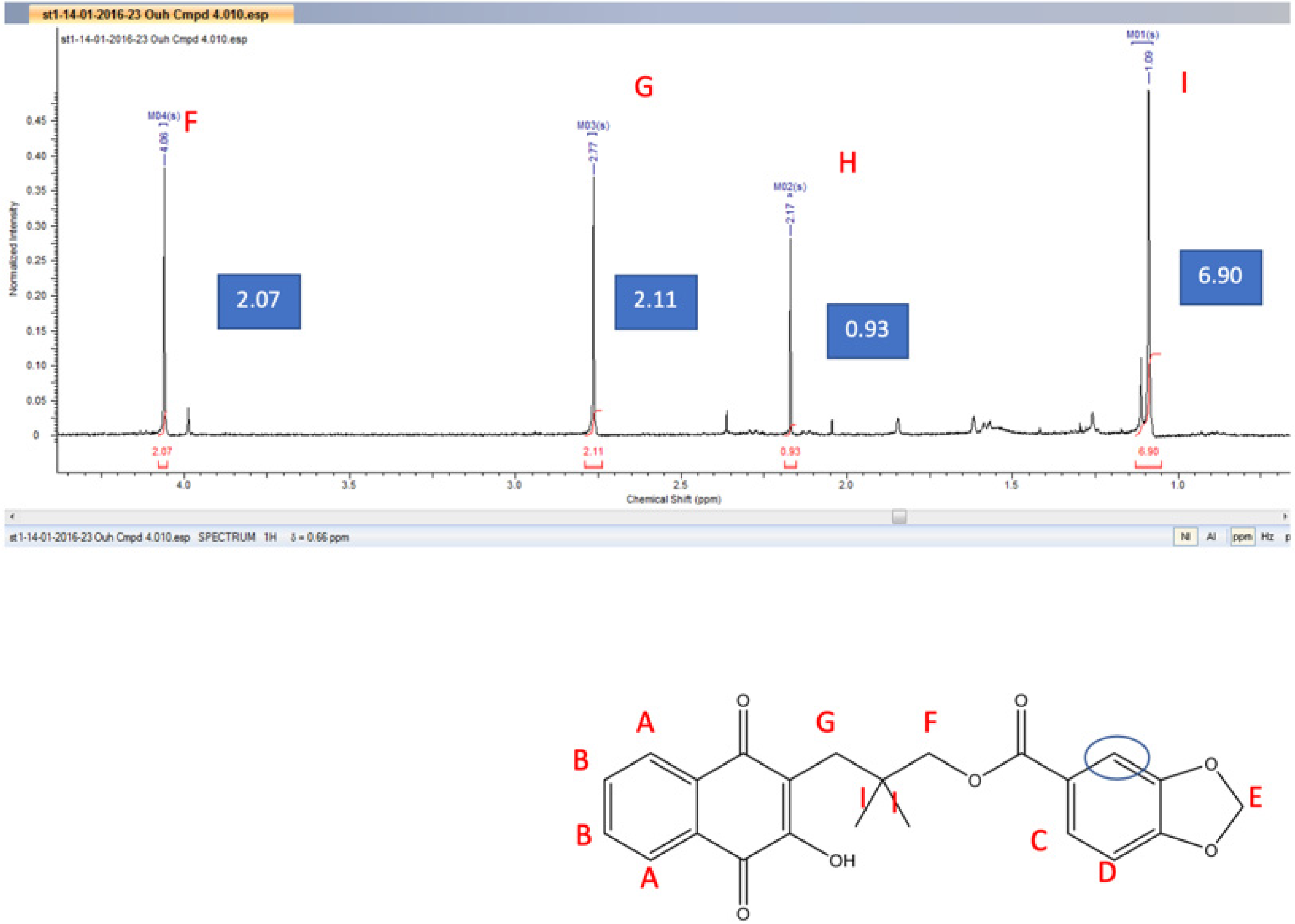

Supplementary Figure S2**D.** ^{1}H NMR spectrum of Rhinacanthin C, highlighting the carbon F,G, H & I of Rhinacanthin D

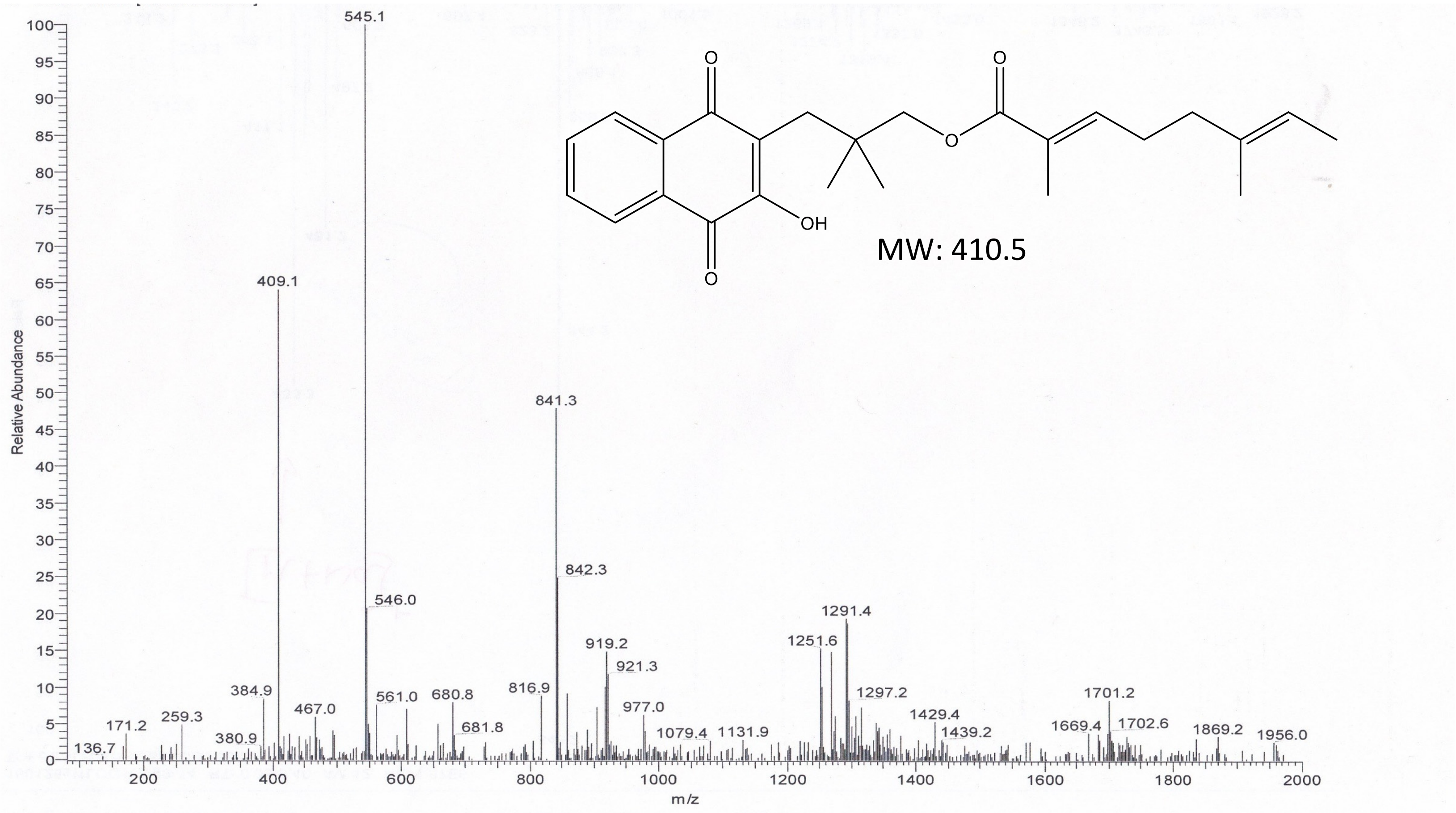

Supplementary Figure S3. Rhinacanthin C Mass Spec. Molecular weight of Rhinacanthin C was determined as 410.5 g/mol.

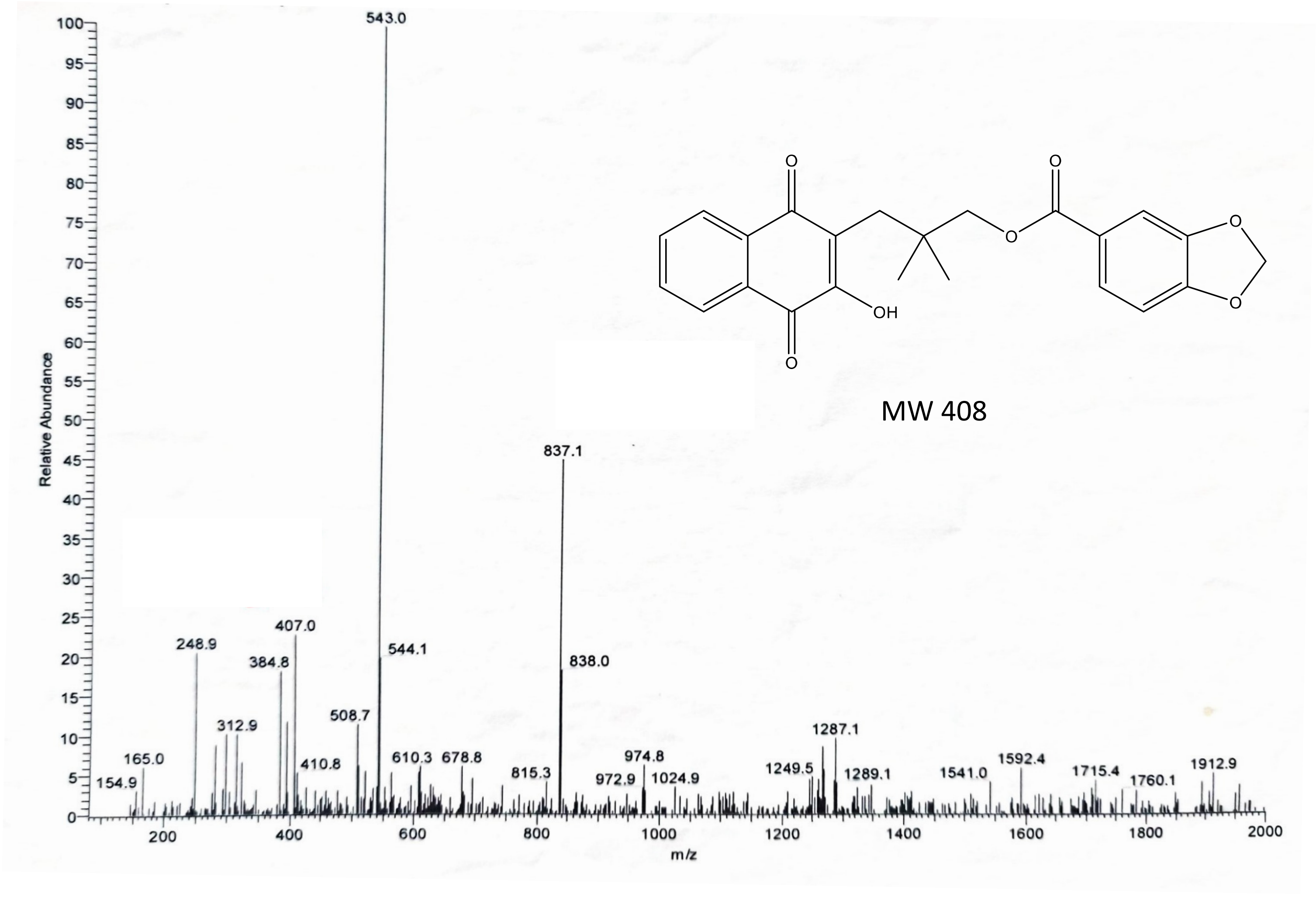

Supplementary Figure S4. Rhinacanthin D Mass Spec. The molecular weight of Rhinacanthin D was determined as 408 g/mol.

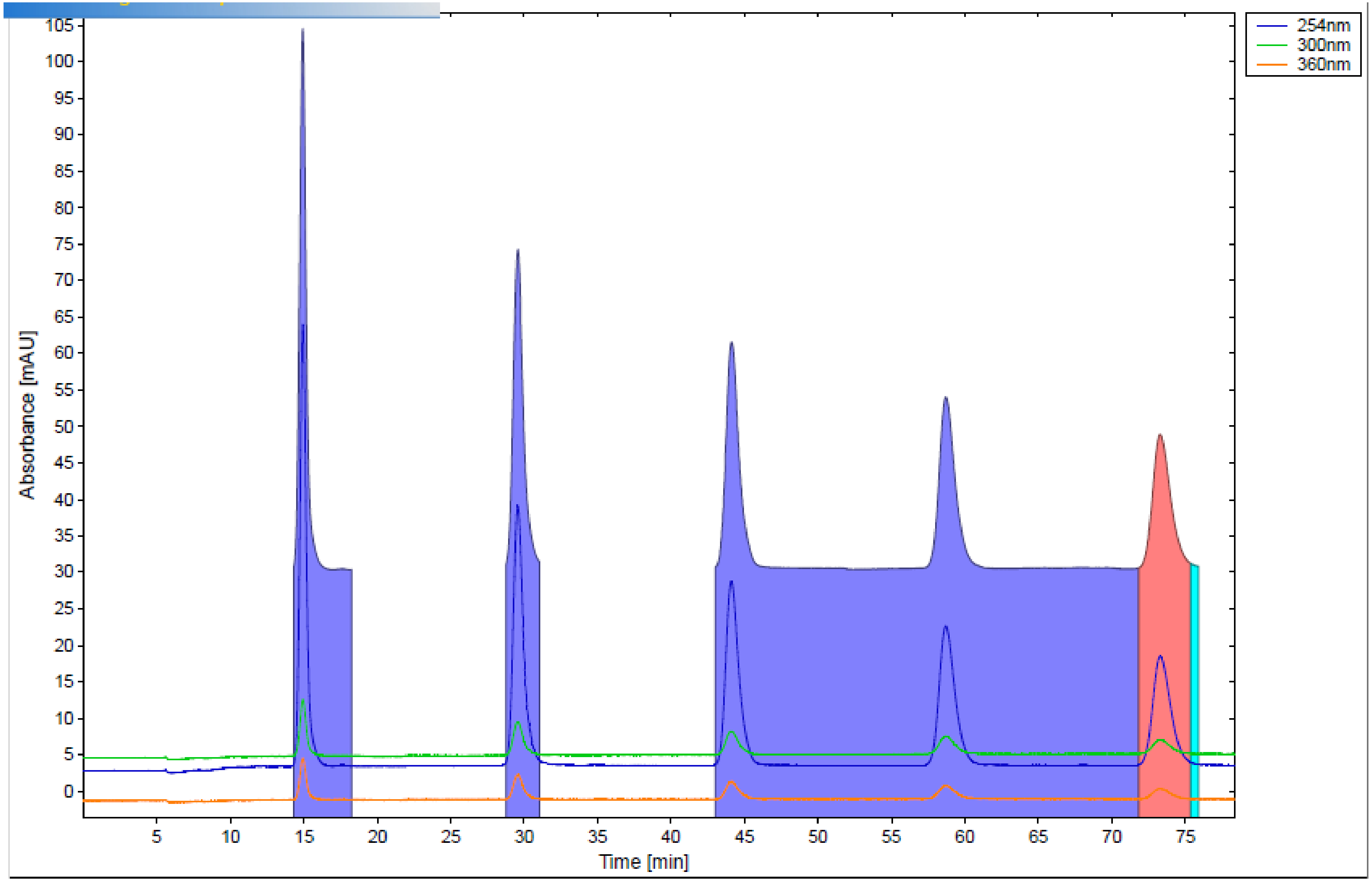

Supplementary figure S5.

Recycling preparative HPLC of Rhinacanthin C, gave repeated symmetrical peaks indicating that the samples were pure for both Rn-C

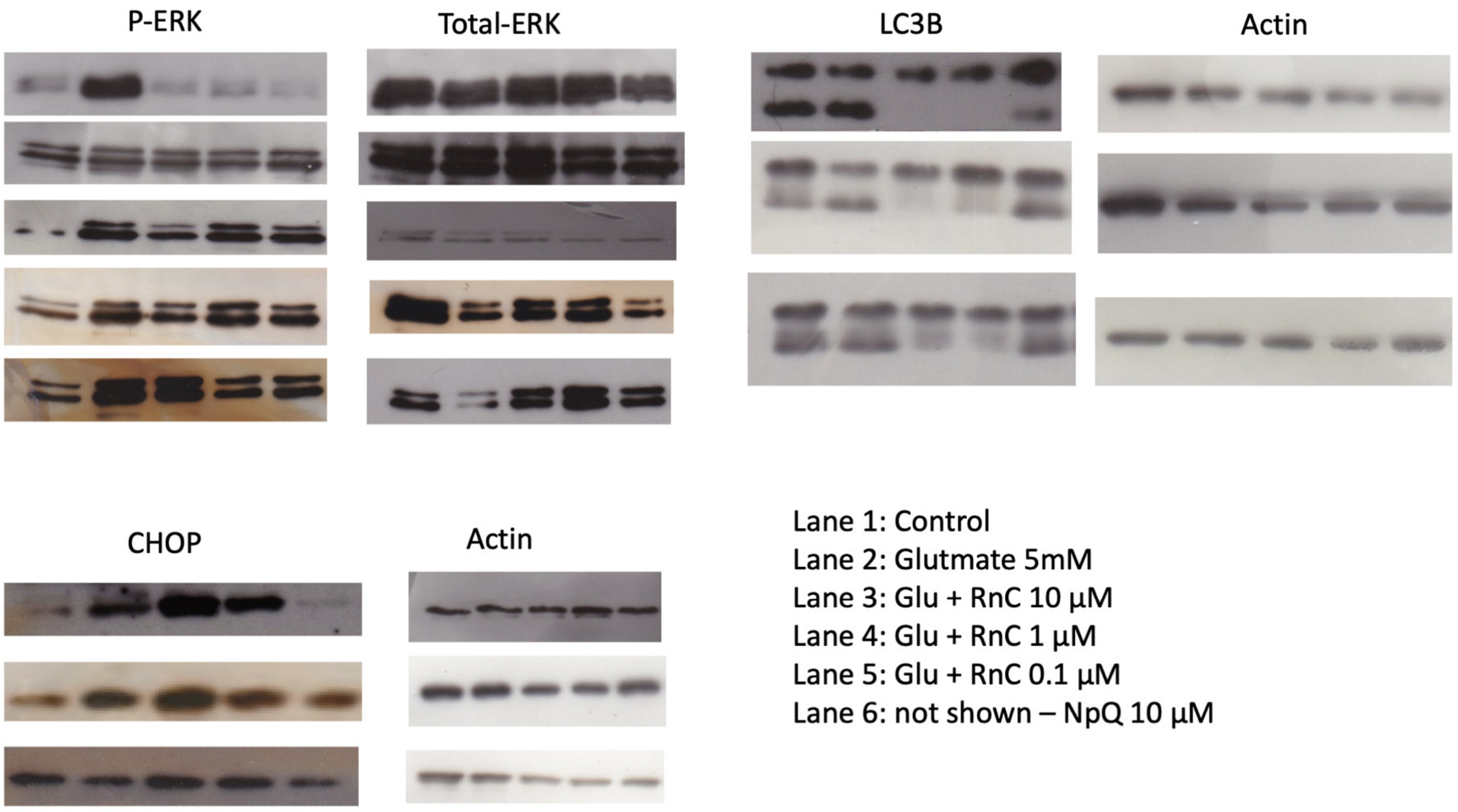

Supplementary Figure S6. All Western blot repeats used for statistical analysis (P-ERK, Total-ERK, LC3B, CHOP and Actin).
